# Orbital Meningoencephalocele and Pulsatile Proptosis: A Rare Entity

**DOI:** 10.7759/cureus.2064

**Published:** 2018-01-15

**Authors:** Muhammad Sohail Umerani, Hira Burhan, Salman Sharif, Tauqir ul Islam, Mehak Hafiz Ghaziani

**Affiliations:** 1 Specialist Neurosurgeon, King Fahd Military Medical Complex, Dhahran; 2 Dow Medical College, Dow University of Health Sciences (DUHS), Karachi, Pakistan; 3 Liaquat National Hospital & Medical College; 4 Department of Oral & Maxillofacial Surgery, Liaquat National Hospital & Medical College

**Keywords:** orbital defects, orbital meningoencephalocele, diplopia, pulsatile proptosis

## Abstract

Orbital roof defects are rare congenital osseous abnormalities that cause protrusion of intracranial contents into the orbit, resulting in a condition known as the orbital meningoencephalocele, a rare cause of pulsatile proptosis. We present a case of a 25-year-old lady, presented to us with complaints of left frontal headache, left eye protrusion and double vision from the left eye for the past three months. Her higher mental functions were intact. Local examination revealed non-axial pulsatile proptosis and an outward depression of the left eyeball along with diplopia. Extraocular movements and pupillary light response were normal with no bruit or orbital tenderness. Computed tomography (CT) scan of the brain and orbit with contrast showed deficient bone on the left orbital roof and floor with left frontal gliotic brain compressing the eyeball. Magnetic resonance imaging (MRI) of the brain and orbit with contrast showed an asymmetrical deformity of the skull and left cerebral hemisphere which was bulging towards the left orbit. We planned a two-staged surgical reconstruction. The orbital roof was first reconstructed using a titanium mesh. Within two weeks of surgery her pulsatile proptosis, diplopia, and headache had considerably improved and the proptosis had resolved with no visible pulsations. She is scheduled for second stage surgery after three months for reconstruction of the orbital floor.

## Introduction

Congenital orbital osseous defects are a rare cause of orbital meningoencephalocele where the intracranial contents protrude through the bony defects into the orbit giving rise to pulsatile proptosis. The defect may be in anywhere in the orbital roof or floor and the medial or lateral orbital wall. Herniation can also be through the natural openings, e.g., the optic foramen or the sphenoid fissure. Intracranial contents passing through these defects give rise to varying degrees of proptosis. Although proptosis is seen in many pathological conditions, the causes of pulsatile proptosis are few and can also occur in some cases of Von Recklinghausen's disease [1]. Apart from the rare congenital nature of the disease, other causes include carotid cavernous fistula (CCF), vascular orbital tumors, and orbital defects seen following cranial surgery and post-traumatic orbital roof fractures. The approach towards surgical management depends upon a substantial radiological evaluation of the orbital defect.

## Case presentation

A 25-year-old female with no known co-morbidities or history of previous illness or trauma presented to the neurosurgery outpatient department. According to her, she was well three months back, when she suddenly developed episodes of severe left frontal headache which were continuous for hours to days. It was throbbing in character not associated with symptoms of nausea, vomiting, dizziness or fits. At many occasions, she complained of double vision with severe headache. For the same length of time, she also noticed that her left eye was bulging outwards along with pulsation in the same protruded eye which gradually increased and since one month it had been so obvious that anyone looking at her could appreciate it.

Her general physical examination was unremarkable. She was conscious and well oriented to time, place and person. Her higher mental functions, gross motor, fine motor and sensory functions were intact. On local examination of the left eye, there was non-axial pulsatile proptosis with outward depression of the left eyeball. On visual acuity, she was assessed to be emmetropic with diplopia in her left eye. Ocular movements in both eyes were normal. There was no tenderness on palpation but pulsations were felt on left eye. No bruit was heard on auscultation.

**Figure 1:**
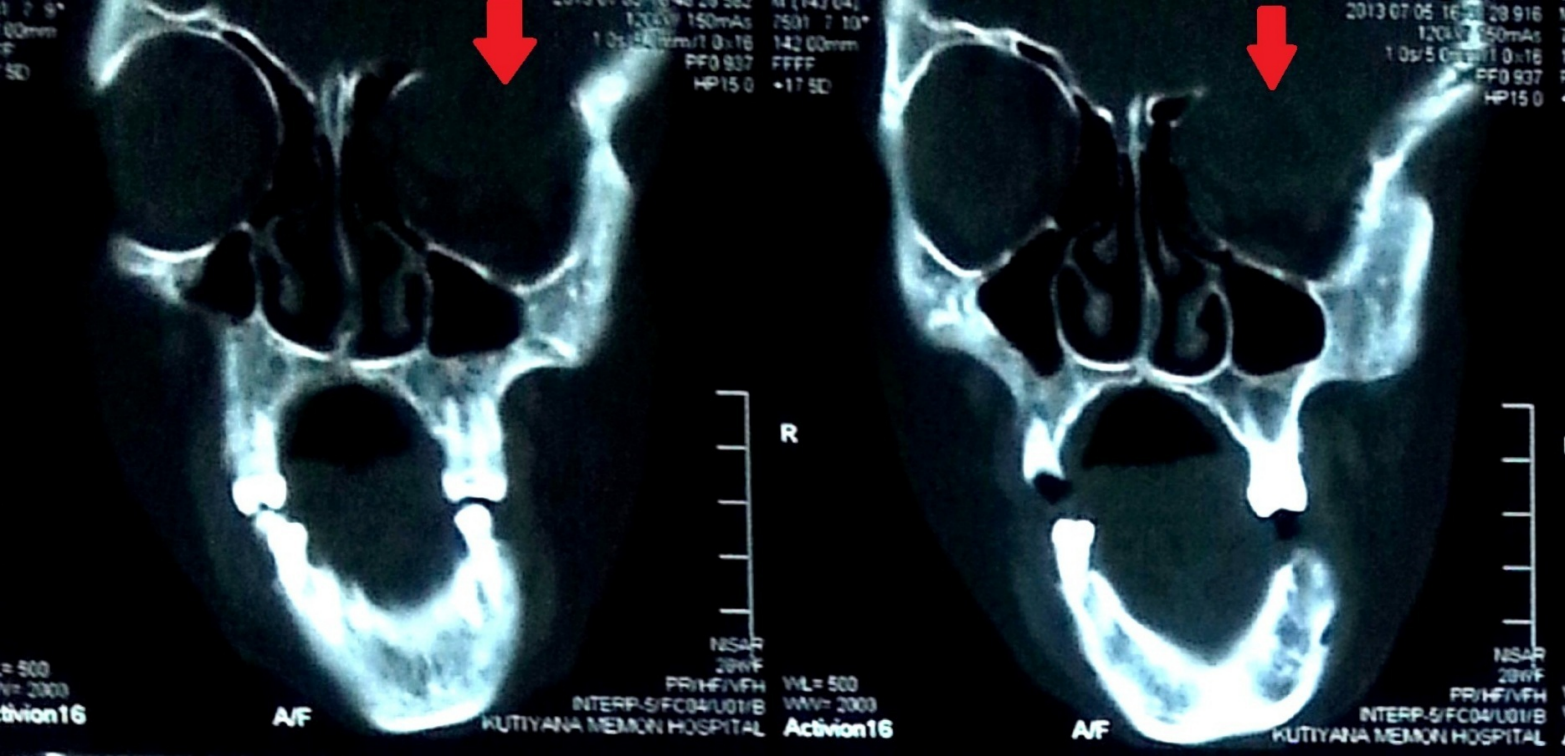
CT scan bone window coronal views showing deficient roof of the left orbit. CT: Computed tomography.

**Figure 2:**
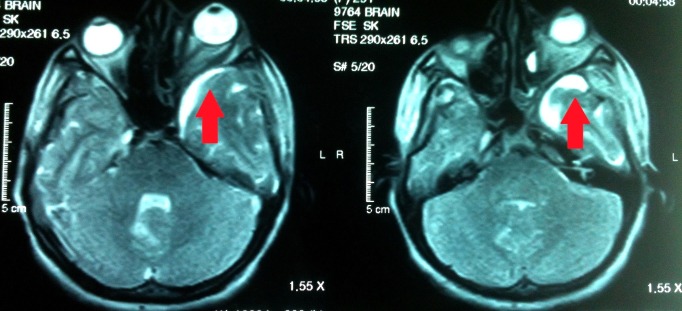
MRI T2WI showing deformity of left cerebral hemisphere causing extrinsic pressure towards left orbit leading to proptosis. MRI: Magnetic resonance imaging; T2WI: T2 weighted image.

Computed tomography (CT) scan of the brain with contrast study showed deficient bone on left orbital roof and floor with left frontal gliotic brain compressing the eyeball causing it to move out and down (Figure 1). Her magnetic resonance imaging (MRI) brain and orbit with contrast study showed an asymmetrical deformity of the skull and left cerebral hemisphere which was bulging towards the left orbit, causing extrinsic pressure resulting in proptosis of the eyeball (Figure 2). It also showed widened cortical sulci due to decreased volume. There was indirect evidence of widening of left lateral ventricle and Sylvian fissure due to hemi-atrophic changes. Contrast studies did not elucidate any abnormal enhancement. Right orbit, right hemisphere, brainstem, and cerebellum appeared normal. There was no evidence of demyelination or any intracranial mass. The affected orbit was larger and downwards than normal. There was an absence of floor and roof of the left orbit.

**Figure 3:**
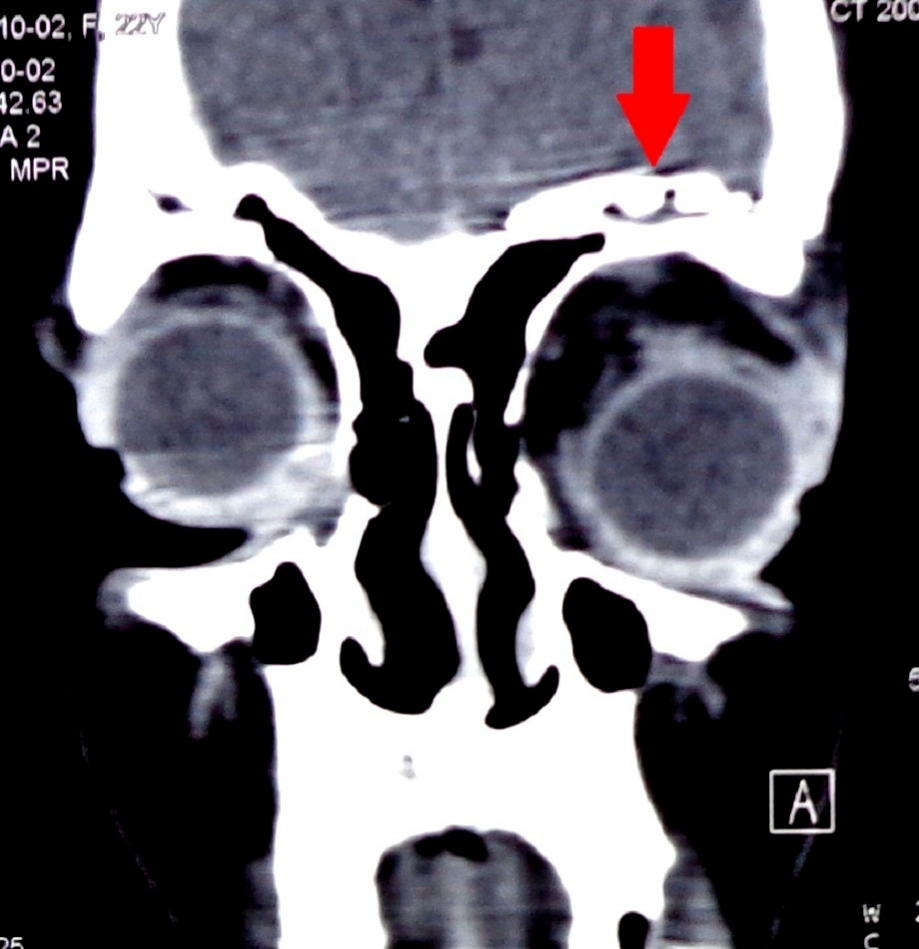
Post-operative CT scan coronal view (bone window) with metallic prosthetic covering the bony defect. CT: Computed tomography.

**Figure 4:**
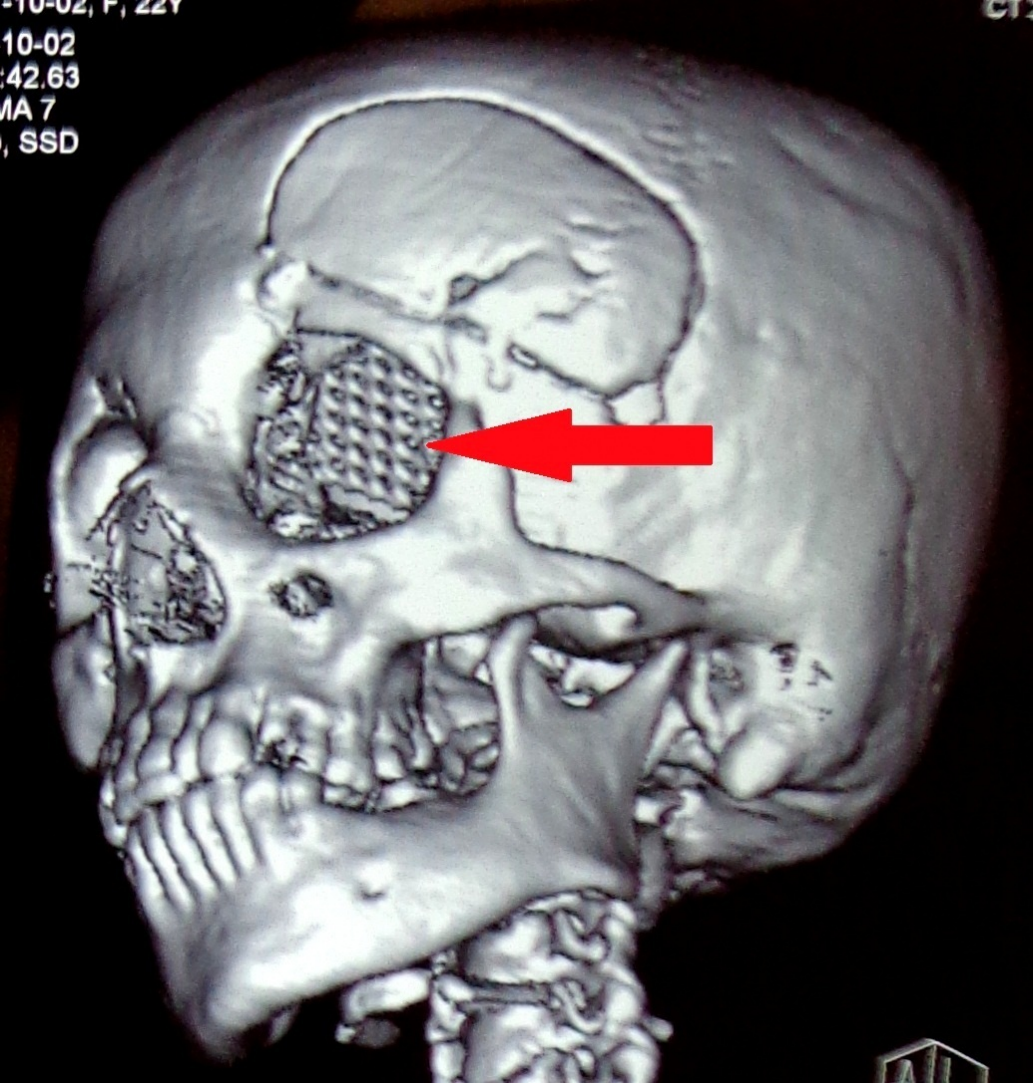
Post-operative CT, 3D reconstruction showing metallic prosthesis seen in roof of the left orbit. 3D: Three-dimensional; CT: Computed tomography.

With this information, a working diagnosis of orbital meningoencephalocele was made and a maxillofacial consult was taken. The patient and her family were counseled regarding the condition and about our plan of surgical intervention that included a two-staged procedure. Left frontal craniotomy was done as a part of the first stage. Per-operatively after opening the dura, frontal lobe was retracted without violation of the arachnoid. The retraction assisted in determining the bony defect. Then a titanium mesh was used to reconstruct the orbital roof and was fixed with screws over the inner surface of superior orbital rim. Duroplasty was performed and the frontal bone flap was secured. A subgaleal drain was then placed and the wound was closed in layers. The drain was removed at the first post-operative day and she was mobilized and discharged on third post-operative day. Her post-operative CT scan with three-dimensional (3-D) reconstruction confirmed the appropriate placement of metallic prosthesis covering the bony defect in the roof of the left orbit (Figures 3-4).

At her two-week follow-up, her diplopia and headache had considerably improved and the proptosis had resolved with no visible pulsations. She is planned for second stage surgery after three months in which we plan to reconstruct the orbital floor.

## Discussion

Proptosis is the abnormal anterior displacement of the eye from the orbit. Although the terms exophthalmus and proptosis are used interchangeably, the former is used for cases due to endocrinopathies. Common etiologies of pulsatile proptosis include CCF, vascular orbital tumors and from transmitted intracranial pulsations through the osseous orbital defects seen either congenitally in neurofibromatosis type I or following cranial surgery and post-traumatic orbital roof fractures. Although it is rare to have a congenital osseous orbital defect, such defect appears to be one of the osseous manifestations of von Recklinghausen's disease [1]. Not only that the orbital osseous defect is a rare congenital entity, protrusion of intracranial contents through such defect is also a very rare cause of proptosis [2]. The rarity of this condition can be appreciated from the fact that scarce literature is present on this topic.

The condition usually develops in infancy and childhood, although it may be evident at birth and rarely delayed until adolescence. Females are affected about twice as often as males. The osseous defect is of two types; anterior (the commonest type) and posterior. Anterior type is located between the frontal, lacrimal, cribriform bone and the nasal process of the maxilla. Sometimes it is evident at the base of the nose. The posterior type is less common. There may be a defect in the roof, floor or the orbital walls causing non-axial proptosis. Small defects are rarely symptomatic. The herniated intracranial content may either be a meningocele, encephalocele, or hydroencephalocele. In the majority of cases the brain substance, which is usually atrophic and oedematous, is incorporated. There may be associated chemosis and edema of the lids especially the upper lid. The proptosis is because of the widening of the ventricle and raised unopposed pressure of the cerebrospinal fluid that gradually pushes the brain forward through the defect, causing it to herniate into the orbit and forcing orbital contents downwards and outwards. The pulsation is due to the direct transmission of the pulsation of the brain to the eyeball. Proptosis usually increases on coughing, bending or straining. Those associated with bruit are usually because of carotid cavernous fistula or congenital arteriovenous malformation seen either in isolation or as part of Wyburn-Mason Syndrome. Rare causes of pulsatile proptosis have been reported in metastatic disease of the orbit from carcinoma of thyroid and renal cell carcinoma [3, 4].

A correct diagnosis is of substantial importance for management. The final diagnosis of the disorder, however, builds upon radiological examination of the skull and brain. The semblance is discrete and not likely to be confused with any other condition. While, bony deformity may vary from case to case, in our patient, there is an asymmetrical deformity of skull and left cerebral hemisphere, which is bulging towards the left orbit, causing extrinsic pressure on the eyeball resulted in proptosis. Large bony defects demand aesthetic skills for closure and require either bone graft, a dural substitute (pericranium or fascia lata), or gel film. Synthetic material like titanium has been used. In this case, we used a titanium mesh to reconstruct the orbital roof although outer calvarial bone grafts have also been used in some cases [5].

It seems probable that some of these cases never develop symptoms which call for treatment and go through life without the condition being recognized. Certainly, the degree of pulsation of the globe in our case was such that there was diplopia and the proptosis was severe.

## Conclusions

Orbital roof defects are rare congenital osseous abnormalities that cause protrusion of intracranial contents into the orbit, resulting in a condition known as the orbital meningoencephalocele, a rare cause of pulsatile proptosis. A correct diagnosis is of substantial importance for management. The final diagnosis of the disorder, however, builds upon radiological examination of the skull and brain. It seems probable that some of these cases never develop symptoms which call for treatment and go through life without the condition being recognized, however, once symptomatic, they require prompt surgical reconstruction. Large bony defects demand aesthetic skills for closure and require either bone graft, a dural substitute (pericranium or fascia lata), or gel film. Synthetic material like titanium mesh can also be used.
